# A nomogram model for predicting prognosis of obstructive colorectal cancer

**DOI:** 10.1186/s12957-021-02445-6

**Published:** 2021-12-02

**Authors:** Jian Lv, Yuan yuan Liu, Yi tao Jia, Jing li He, Guang yao Dai, Peng Guo, Zhao long Zhao, Yan ni Zhang, Zhong xin Li

**Affiliations:** 1 Department of Emergency, Hebei General Hospital, No. 348 Heping West Road, Shijiazhuang, 050051 Hebei China; 2 Department of Anorectal Surgery, Huanghua General Hospital, No. 262 Xinhua Road, Huanghua, 061100 Hebei China; 3 Department of Oncology, Hebei General Hospital, Shijiazhuang, 050051 Hebei China; 4 Second Department of Surgery, The Fourth Hospital of Hebei Medical University, No. 12 Jiankang Road, Shijiazhuang, 050011 Hebei China; 5 Department of Anorectal Surgery, The First Hospital of Shijiazhuang, No. 36, Fanxi Road, Shijiazhuang, 050011 Hebei China; 6 Department of Plastic Surgery, The Fourth Hospital of Hebei Medical University, Shijiazhuang, 050011 Hebei China; 7 Department of Anesthesiology, The Fourth Hospital of Hebei Medical University, No. 12 Jiankang Road, Shijiazhuang, 050011 Hebei China; 8 Department of General Surgery, Shanghai Ninth People’s Hospital, Shanghai Jiao Tong University School of Medicine, Shanghai, 200011 China; 9 Department of General Surgery, The First Affiliated Hospital of Hebei Medical University, No. 89 Donggang Road, Shijiazhuang, 050000 Hebei China

**Keywords:** Colorectal cancer, Obstruction, Prognosis, Nomogram

## Abstract

**Background:**

The prognosis of obstructive colorectal cancer (oCRC) is worse than that of nonobstructive colorectal cancer. However, no previous study has established an individualized prediction model for the prognosis of patients with oCRC. We aimed to screen the factors that affect the prognosis of oCRC and to use these findings to establish a nomogram model that predicts the individual prognosis of patients with oCRC.

**Methods:**

This retrospective study collected data of 181 patients with oCRC from three medical hospitals between February 2012 and December 2017. Among them, 129 patients from one hospital were used as the training cohort. Univariate and multivariate analyses were used in this training cohort to select independent risk factors that affect the prognosis of oCRC, and a nomogram model was established. The other 52 patients from two additional hospitals were used as the validation cohort to verify the model.

**Results:**

Multivariate analysis showed that carcinoembryonic antigen level (*p* = 0.037, hazard ratio [HR] = 2.872 [1.065–7.740]), N stage (N1 vs. N0, *p* = 0.028, HR = 3.187 [1.137–8.938]; N2 vs. N0, *p* = 0.010, HR = 4.098 [1.393–12.051]), and surgical procedures (*p* = 0.002, HR = 0.299 [0.139–0.643]) were independent prognostic factors of overall survival in patients with oCRC. These factors were used to construct the nomogram model, which showed good concordance and accuracy.

**Conclusion:**

Carcinoembryonic antigen, N stage, and surgical method are independent prognostic factors for overall survival in patients with oCRC, and the nomogram model can visually display these results.

## Background

Colorectal cancer (CRC) is one of the main cancers leading to cancer-related deaths worldwide [1, 2]. Intestinal obstruction is a serious complication in patients with CRC and represents an emergency with a high mortality rate in this population [3]. Approximately 20% of patients with CRC show intestinal obstruction at the first diagnosis [4]. In comparison to CRC patients without obstruction, those with obstruction usually show a later clinical stage, a low degree of tumor differentiation after surgery, and a greater likelihood of metastasis. Thus, their long-term survival rate is poor, and their 5-year survival rate is only between 31 and 42% [5–7]. However, only a few studies have evaluated the factors affecting the prognosis of patients with obstructive CRC (oCRC).

In tumor prognosis research, nomogram models employing regression analysis are frequently used. These models are based on multivariate analysis and integrate the results of logistic or Cox regression to a great extent to predict the probability of a certain clinical event in patients along with intuitive graphical presentations. An increasing amount of literature has reported the advantages of these models in predicting tumor recurrence and metastasis, death, and other prognostic outcomes [8, 9]. In comparison to conventional evaluation methods, the nomogram model can produce more accurate and intuitive predictions [10]. However, individualized prediction models for the prognosis of patients with oCRC have not been reported in the literature.

In this study, we developed a nomogram model based on prognostic factors of patients with oCRC to predict the individual survival rate of these patients.

### Patients and methods

#### Patients

**Fig. 1:**
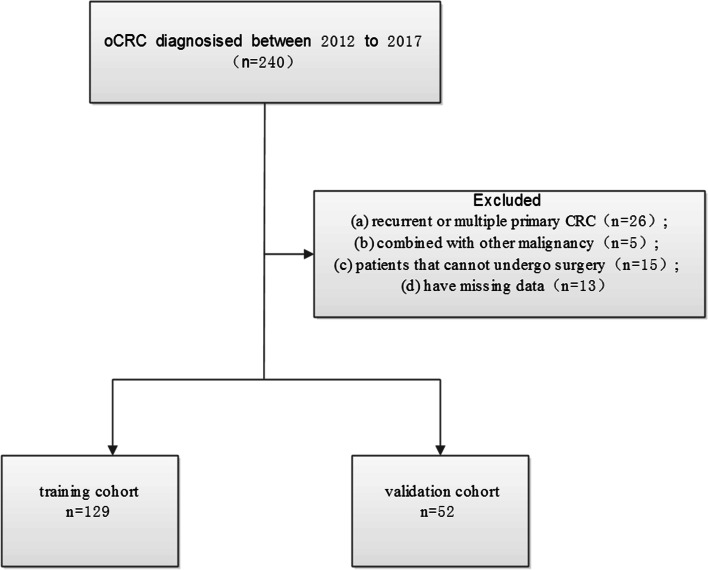
Study flowchart displaying the selection of patients with oCRC according to exclusion criteria. oCRC, obstructive colorectal cancer

Data of 240 patients with oCRC between 2012 and 2017 were retrospectively collected. The inclusion criteria were as follows: primary CRC confirmed by histology, presence of obstruction, and the availability of complete clinicopathological data. Exclusion criteria were (a) recurrent or multiple primary CRCs, (b) other malignant tumors, (c) inability to undergo surgery due to late staging or poor cardiopulmonary function, and (d) inadequate data. A total of 181 cases were eventually included and analyzed in this study (Fig. 1), of which 129 patients from the Fourth Hospital of Hebei Medical University were used as the training cohort for the nomogram, and 52 patients from Hebei General Hospital and Shijiazhuang No. 1 Hospital were used as the validation cohort. The two groups of patients underwent treatment during different periods, i.e., from January 2013 to April 2017 for the training cohort, and from February 2012 to December 2017 for the validation cohort.

#### Surgical procedures

The surgical procedures were conducted in accordance with international guidelines: radical resection was performed by removing the colon or rectal cancer lesions, mesenteric blood vessels and lymphatic vessels responsible for the main blood supply, any organ with direct tumor infiltration, and resectable metastases. The margins of the primary and metastatic lesions were confirmed to be negative. If there was no scope to perform radical resection, palliative resection was performed.

#### Data collection and variables

Clinicopathological information was obtained from the patients’ medical records, including demographics (gender, age), obstruction site, obstruction type, surgery, TNM staging, histological type at diagnosis, white blood cell count (WBC), neutrophil count percentage (NCP), platelet count, and the levels of albumin, carcinoembryonic antigen (CEA), carbohydrate antigen 19-9 (CA19-9), as well as sodium, potassium, calcium, and chloride ions. Cancer staging was based on the 8th edition of the American Joint Committee on Cancer (AJCC)/TNM system.

#### Follow-up

Follow-up assessments of patients were scheduled every 3 months for the first 2 years, every 6 months for the next 3 years, and once a year thereafter. The follow-up assessments included chest and abdominal computed tomography, tumor marker measurements, and endoscopy every 6 months. The last follow-up assessment for the training cohort was performed in May 2017, and the last follow-up assessment for the validation cohort was performed in May 2018. Overall survival (OS) was calculated for the period from the date of treatment initiation to death from any cause. Patients who were lost to follow-up during follow-up or who did not die during the last follow-up were defined as censored.

#### Statistical analysis

SPSS 21.0 was used for statistical analysis. Categorical variables were expressed in percentages (%) and grouped according to clinical regulations. Chi-square and Wilcoxon rank-sum tests were performed to analyze differences between the training cohort and the validation cohort.

Univariate and multivariate Cox proportional hazards models were used to determine potentially important prognostic factors for the entire cohort. The survival curves were plotted using the Kaplan-Meier method, and the log-rank test was used to compare curves. Multivariate Cox regression analysis was performed on the variables that reached the significance level of *p* < 0.05 in the univariate analysis. If a significant effect was observed in the Cox model, independent prognostic factors were determined (*p* < 0.05). The variables of the final model were selected by step-by-step backward regression using the Akaike information criterion.

According to the final Cox proportional hazard regression model and by using the rms package in R version 3.4.2 (http://www.r-project.org/), the nomogram model was constructed. Both internal and external verifications were performed for the nomogram, and the discrimination and calibration of the model were evaluated. The evaluation of discrimination in this article was based on the index of concordance (C-index), i.e., the same number of samples were repeatedly extracted from a given database and then put back, and the internal evaluation of the resolution of the nomogram model was performed in the new sample generated. A C-index of 0.5 indicated that the model had no predictive effect. A C-index of l indicated that the predicted results of the model were completely concordant with the actual results. The closer the C-index was to 1, the better the predicted results of the model. Evaluation of the degree of calibration was based on the calibration plot method, which involved a comparison between the event incidence predicted by the nomogram model and the true incidence. The model aimed to predict the risk value of an event for each patient, arrange these values from low to high, segment the queue, calculate the average predicted risk value (*x*-value) in each segment and the corresponding true risk value (*y*-axis), obtain the calibration point in each segment, and connect the calibration points of each segment to draw the predicted calibration curve. The better the fit between the predicted calibration curve and the standard curve, the better the conformity of the prediction model. The nomogram matches each variable to the corresponding score, and the sum of the scores of all variables is defined as the total score. By drawing a vertical line from the axis of the total score, the estimated survival probability can be obtained, and the principle for predicting the survival probability is based on regression analysis. *p* < 0.05 was considered statistically significant.

## Results

### Patient characteristics and survival

Table 1 lists the demographic and clinicopathological characteristics of the training and the validation cohorts. WBC (*p* = 0.009), M stage (*p* = 0.004), and TNM stage (*p* = 0.002) were significantly different between the training and validation cohorts, which might be attributed to the patients’ hospital preferences. In the training cohort, more than half of the patients were men (*n* = 69, 53.49%). Most of the patients in the two groups were older adults (≥ 60 years old, accounting for 60.47%). Radical resection accounted for 2/3 of the cases (*n* = 86, 66.7%), the most common site of obstruction was the right colon (52, 40.31%), and most patients had chronic incomplete obstruction (120, 93.02%; Table 1). Regarding TNM staging, the proportions of patients with high CEA levels (≥ 5 ng/mL) for each TNM stage were as follows: 50.00% for stage I, 42.22% for stage II, 53.49% for stage III, and 79.49% for stage IV.

**Table 1:** Demographics and pathological characteristics of oCRC patients

Variables		Training cohort		Validation cohort		*p*
All patients		*n*	%	*n*	%	
		129	100	52	100	
Sex	Male	69	53.49	31	59.62	0.453
	Female	60	46.51	21	40.38	
Age (years)	<60	51	39.53	16	30.77	0.269
	≥60	78	60.47	36	69.23	
Obstructive type	Acute complete obstruction	9	6.98	8	15.38	0.079
	Incomplete obstruction	120	93.02	44	84.62	
Obstructive site	Right colon	52	40.31	22	42.31	0.929
	Left colon	49	37.98	20	38.46	
	Rectum	28	21.71	10	19.23	
Surgical procedure	Palliative resection	43	33.33	22	42.31	0.255
	Radical resection	86	66.67	30	57.69	
T stage	T2+3	9	6.98	7	13.46	0.165
	T4	120	93.02	45	86.54	
N stage	N0	60	46.51	33	63.46	0.161
	N1	46	35.66	8	15.38	
	N2	23	17.83	11	21.15	
M stage	M0	90	69.77	47	90.38	0.004
	M1	39	30.23	5	9.62	
TNM stage	I+II	47	36.43	30	57.69	0.002
	III	43	33.33	17	32.69	
	IV	39	30.23	5	9.62	
Histopathology	Adenocarcinoma	100	77.52	41	78.85	0.846
	Mucinous or signet ring adenocarcinoma	29	22.48	11	21.15	
WBC (×10^9/L^)	<9.5	111	86.05	36	69.23	0.009
	≥9.5	18	13.95	16	30.77	
NCP (%)	<75	91	70.54	29	55.77	0.057
	≥75	38	29.46	23	44.23	
HGB (g/L)	>120	62	48.06	29	55.77	0.348
	≤120	67	51.94	23	44.23	
PLT (×10^9/L^)	<350	105	81.4	42	80.77	0.922
	≥350	24	18.6	10	19.23	
CEA (ng/ml)	<5	55	42.64	29	55.77	0.109
	≥5	74	57.36	23	44.23	
CA19-9 (U/ml)	<27	82	63.57	38	73.08	0.221
	≥27	47	36.43	14	26.92	
Albumin (g/L)	>40	56	43.41	25	48.08	0.568
	≤40	73	56.59	27	51.92	
Na (mmol/L)	>137	94	72.87	37	71.15	0.815
	≤137	35	27.13	15	28.85	
K (mmol/L)	>3.5	113	87.6	42	80.77	0.236
	≤3.5	16	12.4	10	19.23	
Ca (mmol/L)	>2.11	114	88.37	41	78.85	0.098
	≤2.11	15	11.63	11	21.15	
Cl (mmol/L)	>99	111	86.05	40	76.92	0.135
	≤99	18	13.95	12	23.08	

In the training cohort, 30 patients died, with a median follow-up of 18 months (range, 1–40 months). In the validation cohort, 16 patients died, with a median follow-up time of 19 months (range, 1–56 months). The 1-year and 3-year OS of the training cohort was 85.0% and 63.5% of that of the validation cohort, respectively, and the OS of the validation cohort was 85.4% and 68.1%, respectively.

### Independent prognostic factors of oCRC

**Fig. 2:**
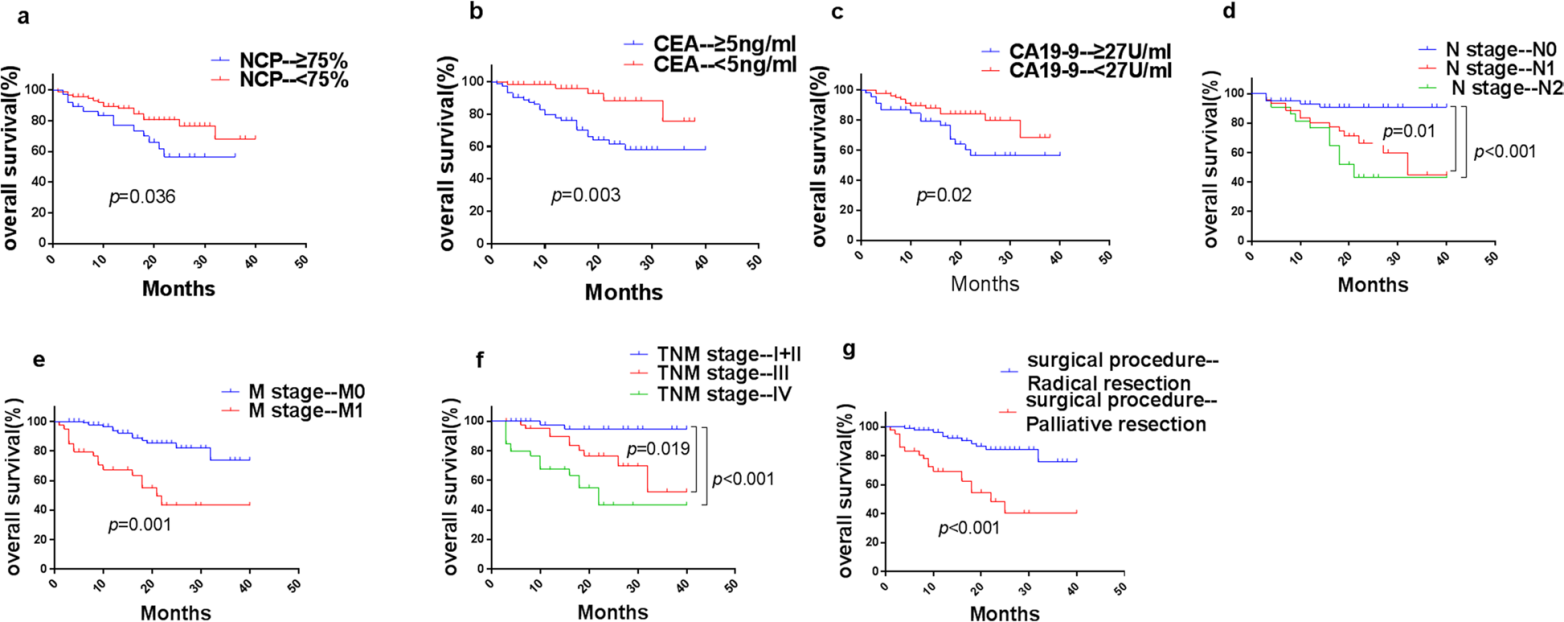
Kaplan-Meier curves of OS for patients with oCRC in the training cohort. **a** NCP, **b** CEA, **c** CA19-9, **d** N stage, **e** M stage, **f** TNM stage, and **g** surgical procedures. CA19-9, carbohydrate antigen 19-9; CEA, carcinoembryonic antigen; NCP, neutrophil count percentage; oCRC, obstructive colorectal cancer; OS, overall survival

Univariate analysis showed that NCP (*p* = 0.036), CEA (p = 0.003), CA19-9 (*p* = 0.02), N stage (*p* < 0.001), M stage (*p* = 0.001), TNM stage (*p* < 0.001), and surgical procedures (*p* < 0.001) were significantly associated with a shorter OS in patients with oCRC (Table 2). However, only CEA (*p* = 0.037, hazard ratio [HR] = 2.872 [1.065–7.740]), N stage (N1 vs. N0, *p* = 0.028, HR = 3.187 [1.137–8.938]; see Fig. 1; N2 vs. N0, *p* = 0.010, HR = 4.098 [1.393–12.051]), and surgical procedures (*p* = 0.002, HR = 0.299 [0.139–0.643]) were shown to be important independent prognostic factors for OS (multivariate Cox ratio) in the univariate risk analysis (Table 2). A survival curve was used to represent risk factors that had a significant impact on prognosis in the univariate analysis (Fig. 2).

**Table 2:** Univariate and multivariate COX regression analyses for OS of oCRC patients in the training cohort

Variables		Univariate analysis	*p*	Multivariate analysis	*p*
		HR (95%CI)		HR (95%CI)	
Sex	Male	1			-
	Female	0.878 (0.428, 1.800)	0.723	-	-
Age	<60	1		-	-
	≥60	1.555 (0.712, 3.398)	0.268	-	-
Obstructive type	Acute complete obstruction	1		-	-
	Incomplete obstruction	2.252 (0.307, 16.536)	0.425	-	-
Obstructive site	Right colon	1		-	-
	Left colon	1.158 (0.518, 2.591)	0.721	-	-
	Rectum	1.099 (0.410, 2.942)	0.851	-	-
Surgical procedure	Palliative resection	1		1	
	Radical resection	0.199 (0.094, 0.422)	<0.001	0.299 (0.139, 0.643)	0.002
T stage	T2+3	1		-	-
	T4	2.229 (0.304, 16.366)	0.431	-	-
N stage	N0	1		1	
	N1	3.820 (1.374, 10.615)	0.01	3.187 (1.137, 8.938)	0.028
	N2	6.582 (2.280, 19.005)	<0.001	4.098 (1.393, 12.051)	0.01
M stage	M0	1		-	-
	M1	3.522 (1.714, 7.239)	0.001	-	-
TNM stage	I+II	1		-	-
	III	6.084 (1.347, 27.475)	0.019	-	-
	IV	14.472 (3.326, 62.961)	<0.001	-	-
Histopathology	Adenocarcinoma	1		-	-
	Mucinous or signet ring adenocarcinoma	1.285 (0.571, 2.894)	0.544	-	-
WBC (×10^9/L)	<9.5	1		-	-
	≥9.5	1.520 (0.619, 3.736)	0.361	-	-
NCP (%)	<75%	1		-	-
	≥75%	2.159 (1.052, 4.432)	0.036	-	-
HGB (g/L)	≤120	1		-	-
	>120	0.617 (0.299, 1.273)	0.192	-	-
PLT (×10^9/L^)	<350	1		-	-
	≥350	1.043 (0.426, 2.555)	0.927	-	-
CEA (ng/ml)	<5	1		1	
	≥5	4.301 (1.643, 11.259)	0.003	2.872 (1.065, 7.740)	0.037
CA19-9 (U/ml)	<27	1		-	-
	≥27	2.356 (1.144, 4.852)	0.02	-	-
Albumin (g/L)	>40	1		-	-
	≤40	0.783 (0.382, 1.602)	0.502	-	-
Na (mmol/L)	>137	1		-	-
	≤137	1.794 (0.853, 3.775)	0.123	-	-
K (mmol/L)	>3.5	1		-	-
	≤3.5	1.048 (0.366, 3.005)	0.93	-	-
Ca (mmol/L)	>2.11	1		-	-
	≤2.11	2.002 (0.818, 4.902)	0.129	-	-
Cl (mmol/L)	>99	1		-	-
	≤99	1.188 (0.454, 3.107)	0.726	-	-

### Nomogram model of oCRC

**Fig. 3:**
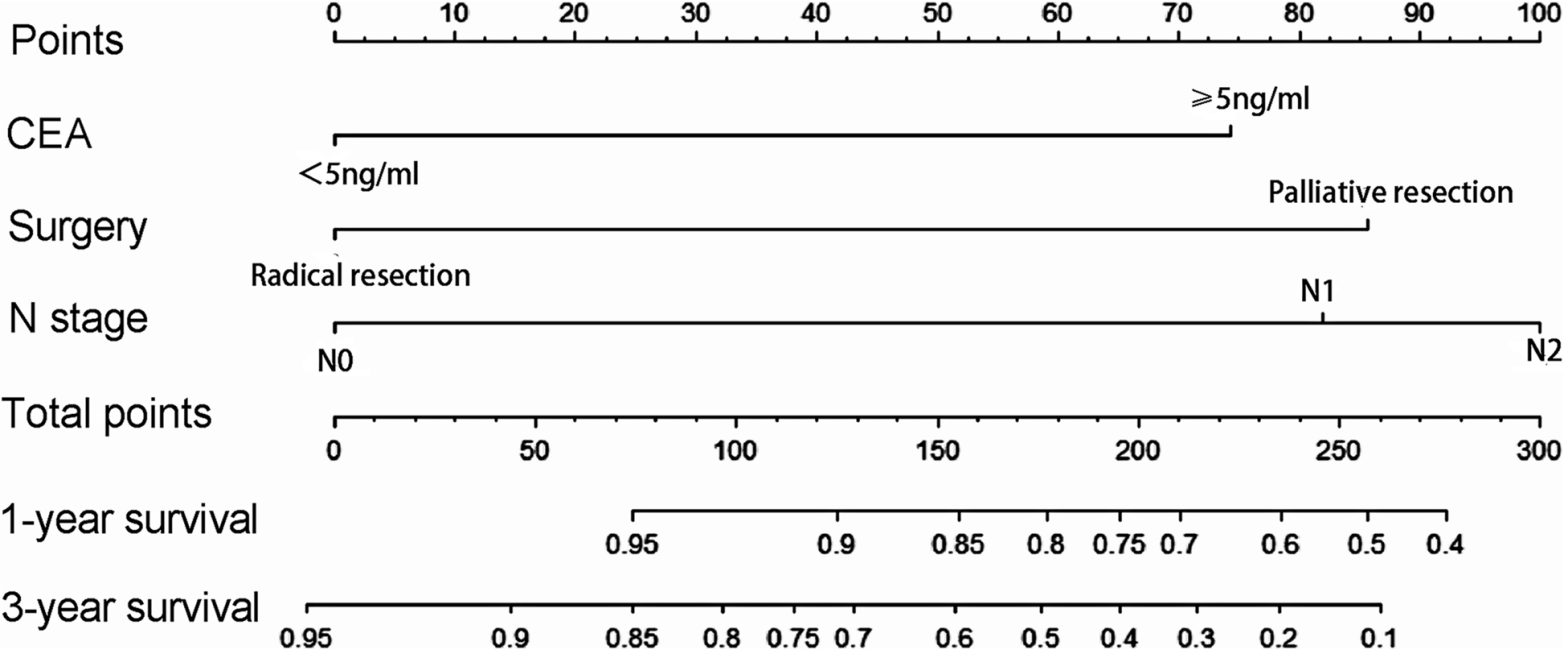
Nomogram model predicting the 1- and 3-year OS in patients with oCRC. The nomogram is used by summing all points identified on the scale for each variable. The total points projected on the bottom scales indicate the probabilities of 1- and 3-year survival. oCRC, obstructive colorectal cancer; OS, overall survival

A nomogram model that included the important predictors in the Cox analysis was established to predict the prognosis of oCRC (Fig. 3). For example, a patient with obstruction had a CEA ≥ 5 ng/ml (74 points), underwent radical surgery (0 points), and postoperative pathology showed no lymph node metastasis (0 points). Thus, the total score is 74 points; the patient’s 1-year survival rate is about 95%, and the 3-year survival rate is about 85%. Had the patient undergone palliative surgery (84 points), the total score would be 158 points. In this case, the patient’s 1-year and 3-year survival rates would be about 84% and 56%, respectively.

### Nomogram model verification

**Fig. 4:**
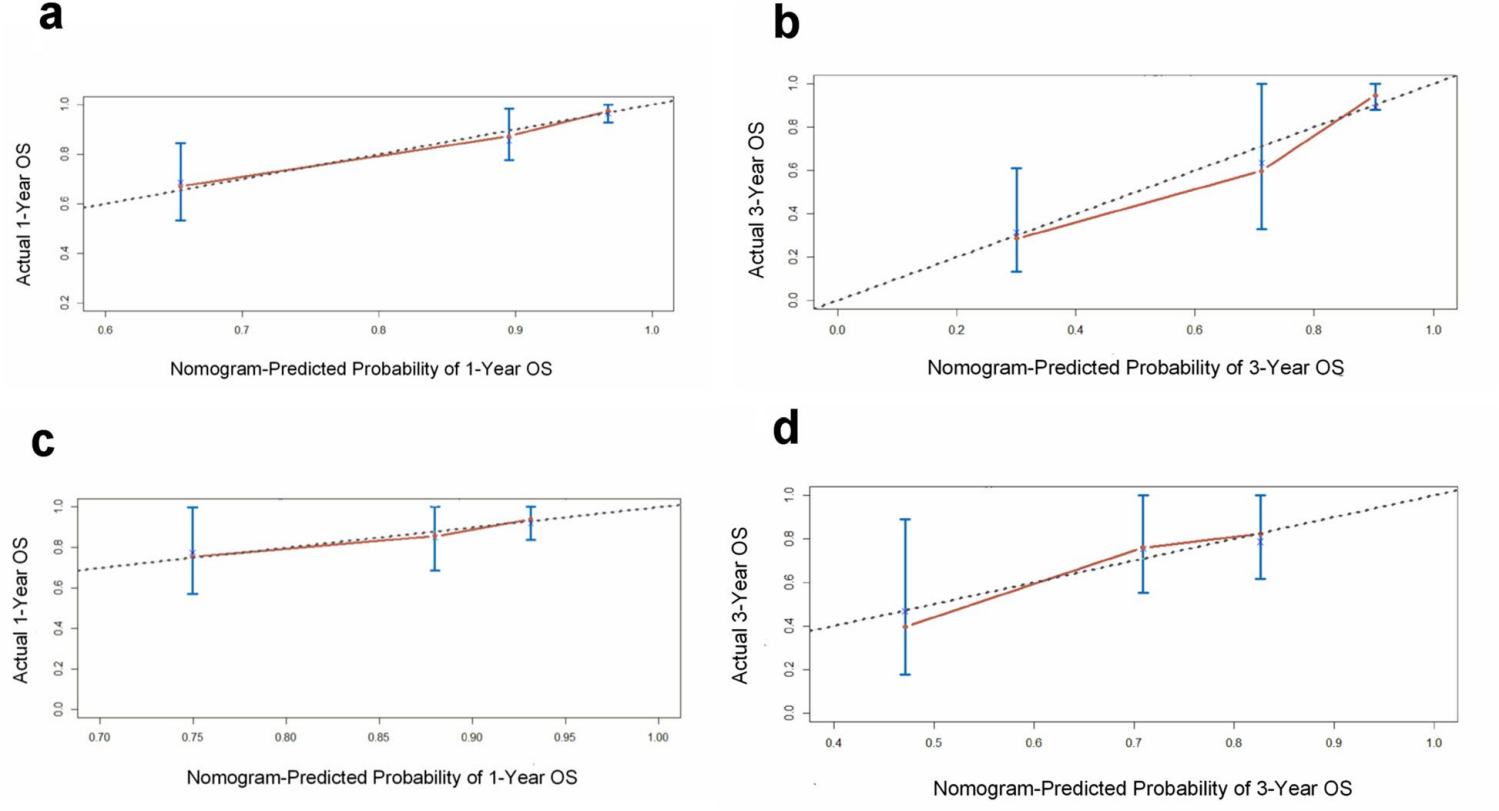
The calibration curves for predicting patient OS at **a** 1 year and **b** 3 years in the internal verification and **c** 1 year and **d** 3 years in the external verification. The OS predicted by the nomogram model is plotted on the *x*-axis, and the actual OS is plotted on the *y*-axis. OS, overall survival

**Fig. 5:**
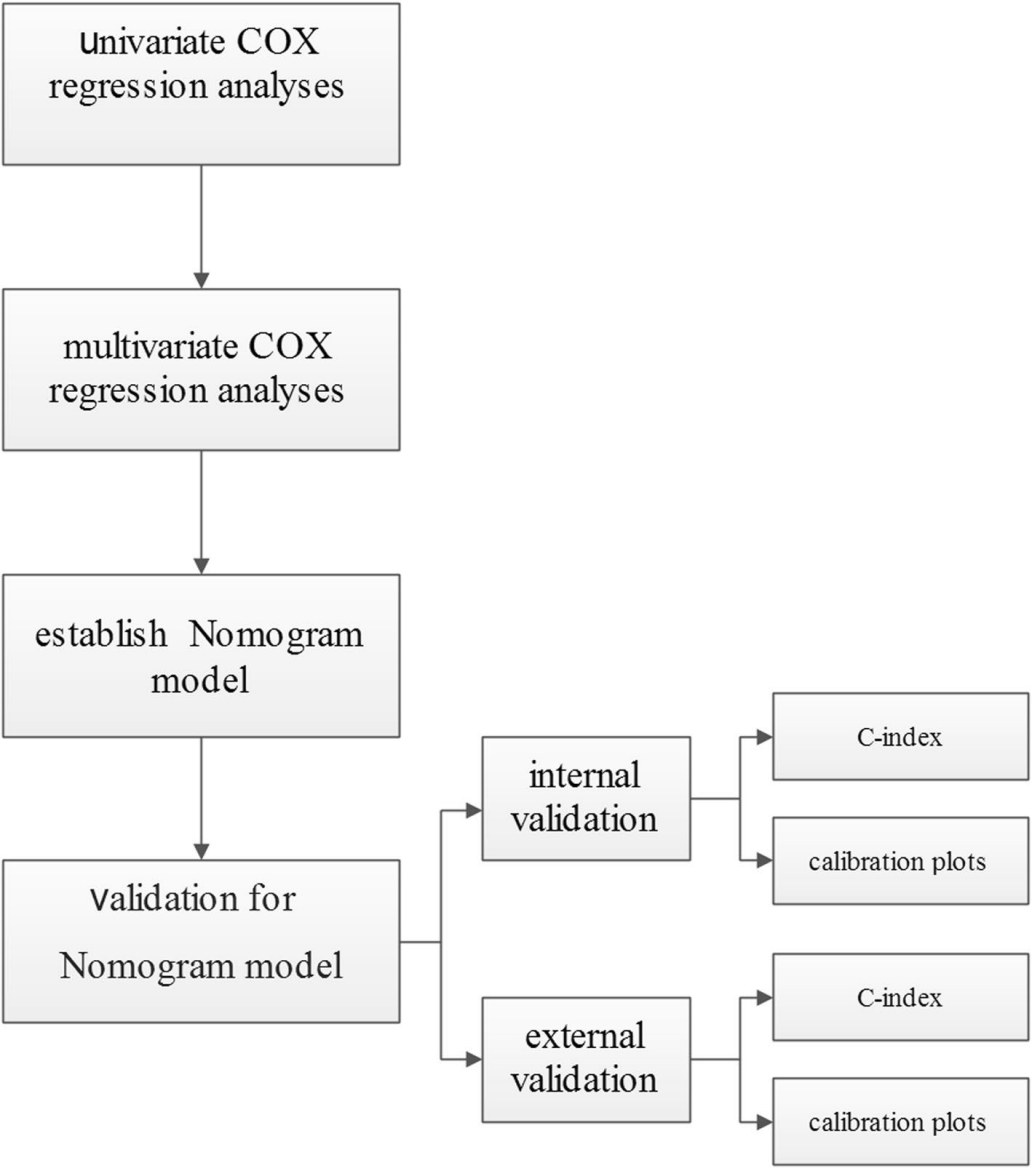
Flowchart displaying the process of building the nomogram

Internal verification showed that the nomogram could accurately predict the C-index of OS, which was 0.797. In the external verification, the C-index was 0.703, showing good concordance. The calibration curve showed that there was good concordance between the predicted and observed values of 1-year and 3-year OS in both training and validation cohorts (Fig. 4). The process followed for building the nomogram is presented as a flowchart in (Fig. 5).

## Discussion

CRC is one of the leading causes of cancer-induced deaths worldwide. The National Comprehensive Cancer Network (NCCN) guidelines show that intestinal obstruction is one of the high-risk factors for recurrence [11]. Colonic obstruction caused by malignant CRC leads to more complicated clinical problems [12, 13]. In comparison to nonobstructive CRC, oCRC is associated with poor postoperative mortality in both short- and long-term follow-up [7, 14]. Mohd Suan et al. reported that intestinal obstruction is related to a low survival rate of patients with CRC [5]. At present, there is no suitable method to predict the prognosis of patients with oCRC. In this study, based on univariate and multivariate COX regression analyses, it was concluded that CEA, stage N, and surgical procedures are independent risk factors for the prognosis of oCRC. Using these risk factors, we developed a nomogram to visualize the results of the regression analysis, and it can also be used to predict the survival probability of patients with oCRC.

Tumor biomarkers have been widely used in the diagnosis and post-treatment follow-up of patients with CRC [15]. The latest research shows that Pre-sarcopenia is a clinical factor significantly associated with OS and DFS in obstructive colorectal cancer [16]. Traditionally, CEA is one of the tumor biomarkers used to predict recurrence, prognosis, and treatment effect in these patients [17, 18]. A high level of CEA usually indicates the possibility of larger tumors, more lymph node metastases, and poor differentiation [19]. As early as 1976, studies by Sugarbaker et al. showed that the CEA level of patients with oCRC before treatment was higher than that of non-oCRC patients [20]. The 5-year disease-free survival rates of CRC patients with normal and elevated CEA concentrations were 84.6% and 69.8%, respectively, whereas the 5-year OS rates were 74.5% vs. 63.4%, respectively [21, 22]. In our study, the results showed that patients with higher CEA levels (≥ 5 ng/mL) had significantly shorter survival. The CEA level had implications in both univariate and multivariate regression analyses and was finally incorporated in the construction of the nomogram model.

The AJCC/TNM staging system remains the basic tool for evaluating the prognosis of patients with CRC. In our study, we analyzed the patients according to the TNM staging system. We could clearly demonstrate that lymph node positivity (N stage) was an independent predictor for worse OS. Enciu et al. reported that patients with oCRC showed a greater incidence of lymph node metastasis [23]. Patients with more lymph node metastases had a worse prognosis, which was concordant between CRC patients with or without bowel obstruction [24]. Distant metastasis is generally considered a sign of poor prognosis in CRC. In our study, the survival period without metastasis was significantly longer only in the univariate, not in the multivariate analysis. This may be attributed to the relatively high censoring rate, which is a limitation of this article.

For patients with oCRC, surgical resection which includes radical resection and palliative resection is a beneficial treatment option [25]. Many early studies on surgical treatment of colorectal cancer suggest that providing radical resection for suitable patients can improve disease-free survival and overall survival [26, 27]. For patients with relatively advanced obstruction who can receive elective surgery, the NCCN guidelines recommend preoperative chemotherapy for advanced colorectal cancer. If the metastatic disease is resectable, surgery is recommended for both primary tumor and metastatic disease [11]. However, some cases of oCRC manifest as an acute colonic obstruction as early as the time of initial visit, and the stage is advanced, making more surgeons choose emergency surgery. Emergency surgery carries a high risk, and this surgical procedure focuses more on safety than radical cure. Compared with elective resection, emergency surgery has several disadvantages, such as increased postoperative morbidity and mortality, higher stoma rate, and lower curative resection rate. Even if the tumor is removed, the effect of this approach on radical cure may be compromised [28–30]. Teixeira et al. discuss the results of emergency surgery for diseases including intestinal obstruction due to colorectal cancer. Their findings indicate that it might be necessary to follow the principle of radical resection in emergency surgery for colorectal cancer [31]. Thus, the use of transitional treatment approaches to solve this problem has been explored in recent years, among which self-expanding metal stents and transanal ileus tubes are the most successful procedures [32, 33]. Research by Tajima showed that preoperative use as a transition to radical surgery could benefit from resection of both the primary tumor and sites of metastasis, which contribute to improved survival [34]. More in-depth, research by Yan and Tajima et al. showed that self-expanding metal stents can better avoid the risks and disadvantages associated with emergency surgery, laparoscopic surgery can be performed in some cases, and the same short-term and long-term prognosis can be obtained as traditional elective open surgery [34, 35]. Okuda et al. suggest that preoperative decompression with an ileus tube results in no significant difference in long-term prognosis compared to emergency surgery, but it can increase the tumor resection rate and the rate of one-stage anastomosis [36]. Actually, no significant differences are reported in oncologic long-term survival between patients undergoing stent placement or decompression tubes as a bridge to surgery and those undergoing emergency surgery. For self-expanding metal stents and decompression tubes, research findings by Suzuki indicate the 3-year DFS rate was significantly higher in the decompression tube group than in the self-expanding metal stents group [37]. When we treat patients with intestinal obstruction due to acute colorectal cancer, we also first deploy stents or ileus tubes for decompression according to the clinical situation of the patient and then complete the radical surgery. In our study, the Kaplan-Meier curve of surgical procedures also shows the advantages of radical resection in patients with oCRC. For patients with advanced oCRC, the use of methods such as ostomy and stent implantation should be considered first to relieve local obstruction, or preoperative chemotherapy should be provided to seek the opportunity for radical resection. At the same time, some preoperative serological examinations can predict the prognosis of patients with obstructive colorectal cancer. The study by Sufana et al. has shown that an increase in preoperative CRP indicates an increase in postoperative complications and encourages preoperative evaluation of inflammatory tendency, which makes preoperative evaluation more adequate [38].

As a prognostic statistical model, a nomogram can not only visually display the relevant indicators that affect the outcome in multifactor regression analyses but also predict the survival probability through a simple graphical representation, making the prediction simpler and more convenient [39, 40]. The construction of the nomogram model in this study is similar to that in many comparable articles. We selected the risk factors determining the prognosis of oCRC patients through univariate and subsequent multivariate Cox regression analyses [41, 42]. The nomogram visualizes the influence of identified risk factors and enables the survival prediction, with the multivariate regression analysis being the core of this model. The results of the internal and external verification show that when predicting the overall survival, the model has high degrees of discrimination and calibrated accuracy. Since the model can predict the risk of death well and is highly consistent with actual incidence data, it has a certain value for clinical applications. However, there are some potential limitations. First, this was a retrospective study with a possible selection bias. Second, the follow-up durations of the training group and the verification group were both very short. Furthermore, the number of follow-up cases was small. Therefore, more patients who received long-term follow-up should be recorded to improve the current nomogram model.

## Conclusions

This study established and verified a nomogram model that can predict the prognosis of patients with oCRC. The nomogram model, which combines CEA expression, N stage, and surgical procedures, was verified internally and externally as a useful tool for risk assessment. Among these three key parameters, only the operation procedure can be controlled by the surgeon. To prolong the survival of patients with oCRC, efforts should be made to change the surgical method from non-radical resection to radical treatment.

## Data Availability

Additional data and materials may be requested from the corresponding author on reasonable request.

## Abbreviations

AJCC: American Joint Committee on Cancer
CA19-9: Carbohydrate antigen 19-9
CEA: Carcinoembryonic antigen
CRC: Colorectal cancer
HR: Hazard ratio
NCCN: National Comprehensive Cancer Network
NCP: Neutrophil count percentage
oCRC: Obstructive colorectal cancer
OS: Overall survival
WBC: White blood cell count
